# Supplementary material for: Effects of Dietary Koumine on Growth Performance, Intestinal Morphology, Microbiota, and Intestinal Transcriptional Responses of Cyprinus carpio
Source: Int J Mol Sci. 2022 Oct 6;23(19):11860. doi: 10.3390/ijms231911860 (PMC9570066; doi:10.3390/ijms231911860)
Supplement: Supplementary file 1 [file ijms-23-11860-s001.zip › Supplemental Table S1 Validation of gene primers sequences..pdf]

Supplemental Table S1. Validation of gene primers sequences.

primers		Sequences (5'→3')
LOC109089219	F	GCTCCATCATCCACCATC
	R	TTACTGCCACCTCCTCTC
LOC109061377	F	AGAGGAAGAAGGAGGAGAATA
	R	AGTGTCAGGAAGGCATCA
acvr1l	F	CGGCTCACTCTACGACTA
	R	CCAGGATGTTCTTGCTCTT
LOC109055285	F	TGAAGCACCACAGGAGAA
	R	AGCAGTATCCACAGCCTT
LOC109098307	F	GCCTTGTATGTGCCTCTG
	R	TCGTCGCTATCTGCTGAT
LOC109058479	F	CCTTCTCTACCACCACTCT
	R	CACCTGTCCTCTCCTTGA
LOC109049104	F	ACGCAGATGGTGTTATGG
	R	ATTGAGACGCCGAGATTG
dusp10	F	ATGACAGATGCCTACAAGTT
	R	TGAGTATGCGTGGAGTGA
grk6	F	AAGGTCGCATTCACAACA
	R	ACTGTAAGAAGCGACTGTAG
LOC109103802	F	CTGTGGCGGAGTATGAAG
	R	CTTGGAGGCTGTTAGTTAGT
LOC109096341	F	GTATTCTGAAGGATGCTGATG
	R	CGTCGTAGTCGGTTGTAAT
LOC109074662	F	GAGAAGGCAGTGAAGGATAA
	R	GTTCTTCTTCCTCTTCCTCTT
LOC109097312	F	GCCGCTGTAATGACCATA
	R	CTCCACTCCACTCCTCTT
LOC109088112	F	AGTAATGATGCCTGTCTGAG
	R	CTCCGTTCGTAGTGATGG

F: (sense primer); R: (antisense primer).
