# Supplementary material for: Effects of Dietary Koumine on Growth Performance, Intestinal Morphology, Microbiota, and Intestinal Transcriptional Responses of Cyprinus carpio
Source: Int J Mol Sci. 2022 Oct 6;23(19):11860. doi: 10.3390/ijms231911860 (PMC9570066; doi:10.3390/ijms231911860)
Supplement: Supplementary file 1 [file ijms-23-11860-s001.zip › Supplemental Table S2 Primers sequences for qRT-PCR..pdf]

Supplemental Table S2. Primers sequences for qRT-PCR.

primers		Sequences (5'→3')
tgfbr1	F	GCTCCATCATCCACCATC
	R	TTACTGCCACCTCCTCTC
acvr1l	F	CGGCTCACTCTACGACTA
	R	CCAGGATGTTCTTGCTCTT
rreb-1	F	ACGCAGATGGTGTTATGG
	R	ATTGAGACGCCGAGATTG
stat5b	F	GTGTGAGAAGTTGGCTGAT
	R	TGGTGACGAGTGCTGATA
smad4	F	AGTAATGATGCCTGTCTGAG
	R	CTCCGTTCGTAGTGATGG
cbp	F	AGAGGAAGAAGGAGGAGAATA
	R	AGTGTCAGGAAGGCATCA
c-fos	F	CTGTGGCGGAGTATGAAG
	R	CTTGGAGGCTGTTAGTTAGT
β-actin	F	GAGGTGGAGGCTAACAAC
	R	GATGTGAGGAGAGGATTCG

F: (sense primer); R: (antisense primer).
