# Supplementary material for: Effects of Dietary Koumine on Growth Performance, Intestinal Morphology, Microbiota, and Intestinal Transcriptional Responses of Cyprinus carpio
Source: Int J Mol Sci. 2022 Oct 6;23(19):11860. doi: 10.3390/ijms231911860 (PMC9570066; doi:10.3390/ijms231911860)
Supplement: Supplementary file 1 [file ijms-23-11860-s001.zip › Supplemental Table S3 Statistics of trimed sequences.pdf]

Supplemental Table S3. Statistics of trimed sequences

Sample	Sequences	Bases(bp)	Average Length(bp)
A1	30612	11464621	374.51
A2	30231	11317206	374.36
A3	30094	11274417	374.64
B1	30512	11439343	374.91
B2	35232	13185850	374.26
B3	43876	16440933	374.71
C1	56546	21183553	374.63
C2	30084	11269310	374.59
C3	30291	11348600	374.65
D1	49901	18683086	374.4
D2	30598	11468203	374.8
D3	30780	11532143	374.66

Note: Sample A: 0 mg/kg; sample B: 0.2 mg/kg; sample C: 2 mg/kg; sample D: 20 mg/kg
