# Supplementary material for: Effects of Dietary Koumine on Growth Performance, Intestinal Morphology, Microbiota, and Intestinal Transcriptional Responses of Cyprinus carpio
Source: Int J Mol Sci. 2022 Oct 6;23(19):11860. doi: 10.3390/ijms231911860 (PMC9570066; doi:10.3390/ijms231911860)
Supplement: Supplementary file 1 [file ijms-23-11860-s001.zip › Supplemental Table S5 Transcriptome sequencing quality.pdf]

Supplemental Table S5. Transcriptome sequencing quality control data statistics

Sample	Clean reads	Clean bases	Error rate(%)	Q20(%)	Q30(%)	GC content(%)
A1	53395918	7810671814	0.0243	98.31	94.82	45.41
A2	49777160	7287546772	0.0246	98.19	94.57	45.94
A3	52591072	7666526295	0.0243	98.31	94.85	45.21
B1	50459784	7430486482	0.0245	98.24	94.62	45.76
B2	61337086	9019606825	0.0238	98.54	95.33	48.98
B3	47470704	6944235669	0.0248	98.09	94.37	45.53
C1	48364510	7038930782	0.0245	98.22	94.62	45.63
C2	58986576	8648127774	0.0238	98.52	95.32	47.02
C3	58059956	8531473814	0.024	98.44	95.09	48.07
D1	51487352	7527543877	0.0241	98.38	95.02	47.25
D2	52096698	7701634147	0.0243	98.33	94.84	47.38
D3	62952880	9240758847	0.0241	98.41	94.99	47.95

Note: Sample A: 0 mg/kg; sample B: 0.2 mg/kg; sample C: 2 mg/kg; sample D: 20 mg/kg; Each group has three replicates (n = 3 for RNA-seq).
