# Supplementary material for: Effects of Dietary Koumine on Growth Performance, Intestinal Morphology, Microbiota, and Intestinal Transcriptional Responses of Cyprinus carpio
Source: Int J Mol Sci. 2022 Oct 6;23(19):11860. doi: 10.3390/ijms231911860 (PMC9570066; doi:10.3390/ijms231911860)
Supplement: Supplementary file 1 [file ijms-23-11860-s001.zip › Supplemental Table S6 Database comment information..pdf]

Supplemental Table S6. Database comment information.

	Expre_Gene number （percent）	Expre_Transcript number （percent）	All_Gene number （percent）	All_Transcript number （percent）
GO	37374(0.6715)	67323(0.6179)	42060(0.6394)	78630(0.5989)
KEGG	34420(0.6184)	69888(0.6414)	38844(0.5905)	82500(0.6284)
COG	47116(0.8465)	94555(0.8678)	52313(0.7952)	110801(0.844)
NR	51044(0.9171)	101171(0.9285)	58034(0.8822)	119866(0.913)
Swiss-Prot	43258(0.7772)	88248(0.8099)	47728(0.7255)	103309(0.7869)
Pfam	39275(0.7056)	82769(0.7596)	41742(0.6345)	95252(0.7255)
Total_anno	51103(0.9181)	101316(0.9298)	58119(0.8835)	120039(0.9143)
Total	55661(1.0)	108962(1.0)	65785(1)	131286(1)
