# Supplementary figures and images for: Effects of Dietary Koumine on Growth Performance, Intestinal Morphology, Microbiota, and Intestinal Transcriptional Responses of Cyprinus carpio
Source: Int J Mol Sci. 2022 Oct 6;23(19):11860. doi: 10.3390/ijms231911860 (PMC9570066; doi:10.3390/ijms231911860)

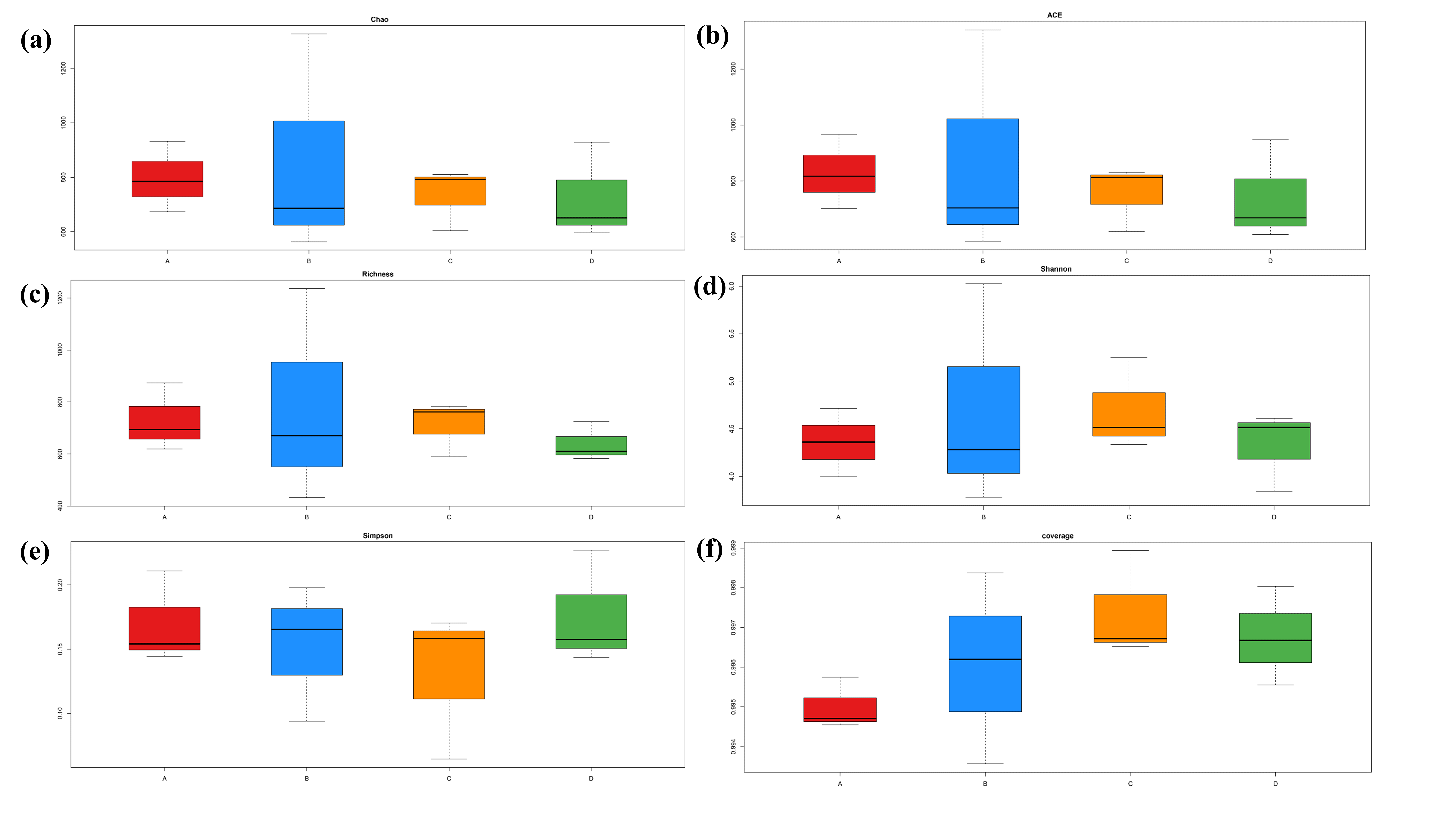

Supplement: Supplementary file 1 [file ijms-23-11860-s001.zip › Supplemental Figure S2.tif]

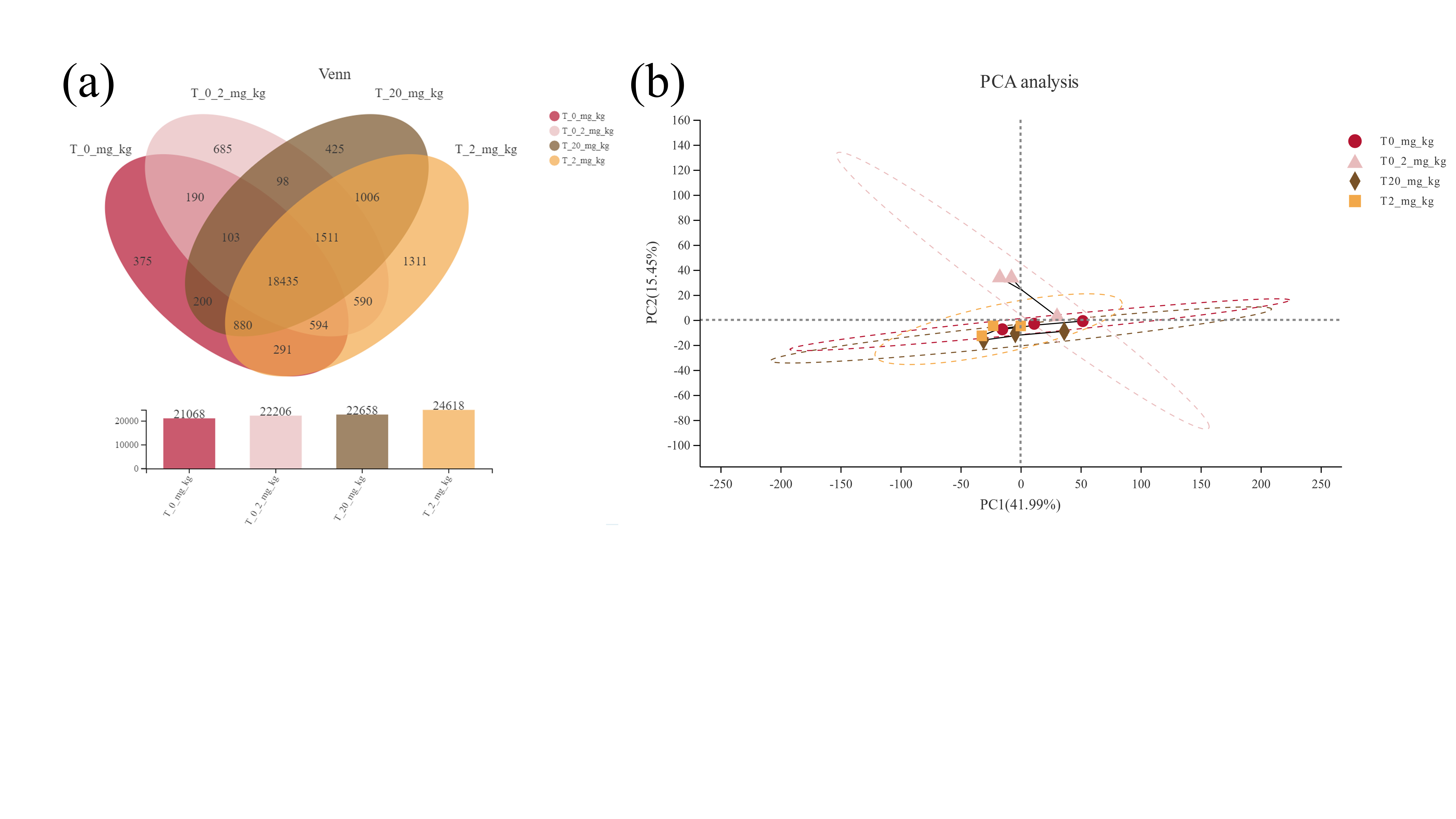

Supplement: Supplementary file 1 [file ijms-23-11860-s001.zip › Supplemental Figure S3.TIF]
